# Reduction of Foveal Avascular Zone After Vitrectomy Demonstrated by Optical Coherence Tomography Angiography

**DOI:** 10.7759/cureus.13757

**Published:** 2021-03-07

**Authors:** Petros Petrou, Constantine D Angelidis, Konstantinos Andreanos, Menelaos Kanakis, Stylianos Kandarakis, Aristotelis Karamaounas, Evangelia Papakonstantinou, Nikolaos Mamas, Konstantinos Droutsas, Ilias Georgalas

**Affiliations:** 1 Ophthalmology, "G. Gennimatas" General Hospital of Athens, Athens, GRC; 2 Ophthalmology, "G. Gennimatas" General Hospital of Athens, National and Kapodistrian University of Athens School of Medicine, Athens, GRC; 3 Ophthalmology, National and Kapodistrian University of Athens School of Medicine, Athens, GRC

**Keywords:** vitrectomy, faz, oct, angiography, retina

## Abstract

Purpose: To investigate the effect of pars plana vitrectomy on foveal circulation, and in particular the foveal avascular zone (FAZ), using optical coherence tomography angiography (OCTA).

Methods: This was a prospective, non-randomized, comparative case series of patients that underwent vitrectomy. Twenty-six eyes of 26 patients that underwent vitrectomy were studied postoperatively by OCTA. Our patients underwent 23 or 25G pars plana vitrectomy (PPV) for any posterior segment pathology. Three-dimensional OCTAs (DRI Triton Swept Source OCT; Topcon) of the capillary plexus were obtained three months post-operatively. The FAZ measurements of the fellow eyes were used as controls.

Main outcome measures: Change in FAZ area between vitrectomized eyes and controls.

Results: From a total of 26 patients, 17 underwent vitrectomy due to retinal detachment (RD). Almost all patients demonstrated a statistically significant reduction in FAZ size based on the OCTA measurements. Τhe mean difference in FAZ size for the superficial capillary plexus (SCP) was -93.77 ± 71.73 μm and for the deep capillary plexus (DCP) -88.87 ± 75.41 μm, both statistically significant (p=0.000), while the amount of reduction in μm was the same for both SCP and DCP.

Conclusion: The foveal avascular zone seems to be reduced following vitrectomy as shown by optical coherence tomography angiography. It is postulated that this may be the result of changes in the physiology of the vitrectomized eye, and that this change should be attributed to the removal of the vitreous itself rather than other structures such as the internal limiting membrane.

## Introduction

Since the introduction of pars plana vitrectomy (PPV) by Robert Machemer in 1971 [1] there have been continuing refinements of the posterior segment surgical techniques as well as an increasing experience in the management of posterior segment disease. A significant number of clinical entities have been added to the indication list for vitreous surgery over the years. However, the exact pathophysiological mechanisms by which the removal of vitreous affects the different anatomical and functional structures of the eye have not been adequately elucidated in the literature to date.

The physiology of vitrectomy remains under investigation and the full understanding of it may facilitate future research. It is known that while the removal of vitreous increases the risk of cataract formation and iris neovascularization, it decreases the risk of retinal neovascularization and macular edema [2]. The circulation of oxygen and cytokines is improved following vitrectomy [2] and PPV has been reported to improve blood flow velocity in eyes with diabetic cystoid macular edema [3]. Moreover, it was demonstrated that macular leakage and pooling of fluorescein (during fluorescein angiography) in the cystoid spaces are reduced in eyes that have undergone surgery with successful resolution of macular edema in diabetic patients.

The recent introduction of optical coherence tomography angiography (OCTA) offers an in situ representation of the retinal and choroidal circulation as a non-invasive procedure [4-6], thus enabling physicians to perform this examination in a safe way in an effort to document the effect of different therapeutic approaches on the foveal microstructure. Novel findings from OCTA have been reported in the literature regarding a variety of posterior segment pathologies such as diabetic retinopathy [7], choroidal neovascularization [8], macular hole [9], and others. However, the effect of PPV on the foveal avascular zone (FAZ) has not been demonstrated with the use of OCTA.

The purpose of the present study was to investigate the role of pars plana vitrectomy in the foveal microcirculation in a cohort of patients with variable posterior segment disease. Optical coherence tomography angiography was used to document the foveal microcirculation in our patients.

## Materials and methods

Institutional approval was obtained and the study procedures conformed to the tenets of the Declaration of Helsinki. We conducted a prospective, non-randomized, comparative case series of 26 eyes of 26 consecutive patients who underwent vitrectomy. All eyes that underwent PPV for any posterior segment pathology and had no ocular pathology in the fellow eye were included in the analysis. Eyes with previous intraocular surgery, high myopia or difficulty in co-operation were excluded from the analysis. Informed consent was obtained from all patients.

Data were collected on demographic characteristics, the pre and post-operative best-corrected visual acuity and the indication for surgery. The patients underwent measurements in both eyes with the optical coherence tomography angiography (DRI Triton Swept Source OCT; Topcon, Newbury Berkshire, UK). Three-dimensional OCT angiograms were obtained over a 3x3 mm and 6x6 mm square on the deep and superficial plexus. The measurements were performed by two investigators who were masked to the indication for surgery and results and the area of the FAZ was determined using the built-in software. If there was more than a 5% difference in the measurements between the two investigators, a third one completed the analysis. The FAZ measurements of the fellow eyes were used as controls.

The OCTA scans were performed on month three post-operatively. All patients underwent 23 or 25 gauge vitrectomy and additional procedures were performed as per indication (e.g. cryotherapy or endolaser retinopexy, gas tamponade, internal limiting membrane peeling - only in cases with macular hole, epiretinal membrane peeling, etc.).

Statistical analysis

Parametric statistical tests depend on the assumption that data are sampled from Gaussian distributions. We analyzed quantitative variables (FAZ size, FAZ size difference between operated and healthy eye, and the difference in percentages). Between them only the FAZ size of the superficial capillary plexus (SCP) and the differerence in FAZ size for the deep capillary plexus (DCP) (both absolute and percentage) followed a normal distribution, according to the Shapiro-Wilk test for composite normality.

In the parametric scenario (comparison of means), Student’s t-test was utilized, while under the non-parametric scenario (comparison of medians), Wilcoxon signed-rank test was applied. Statistical significance was set at p-value < 0.05. Statistical analysis was held in Statistical Package for Social Sciences (SPSS) version 20 (IBM Corp., Armonk, NY, USA).

## Results

Baseline characteristics are shown in Table 1. The indications for surgery included retinal detachment (macula on and macula off), macular hole, epiretinal membrane, vitreous haemorrhage due to retinal break and diagnostic vitrectomy due to vitritis.

**Table 1:** Superficial (SCP) and Deep (DCP) Capillary Plexus Avascular Zone RD: Retinal Detachment; mac off/on: macula off/on; ERM: Epiretinal Membrane; VH: Vitreous Hemorrhage; Vit: Vitrectomy

CASE	DISEASE	SURGERY	SCP VITRECTOMY ^(^^μ^^m^^2^^)^	SCP HEALTHY ^(^^μ^^m^^2^^)^	SCP DIFFERENCE (%)	DCP VITRECTOMY ^(^^μ^^m^^2^^)^	DCP HEALTHY ^(^^μ^^m^^2^^)^	DCP DIFFERENCE (%)
1	RD	Vit	194.950	244.336	-49.386 (-20.21)	255.365	298.363	-42.998 (-14.41)
2	RD	Vit	473.906	550.195	-76.289 (-13.86)	492.138	579.258	-87.120 (-15.04)
3	RD	Vit	207.070	326.162	-119.092 (-36.51)	312.734	344.365	-31.631 (-9.18)
4	RD mac off	Vit	124.102	208.125	-84.023 (-40.37)	217.529	364.131	-146.602 (-40.26)
5	RD	Vit	103.535	166.025	-62.490 (-37.63)	147.041	220.693	-73.652 (-33.37)
6	RD plus ERM	Vit	62.051	157.500	-95.449 (-60.60)	240.732	528.047	-287.315 (-54.41)
7	RD plus ERM	Vit	53.448	227.461	-174.013 (-76.50)	243.281	356.484	-113.203 (-31.75)
8	RD plus ERM	Vit	73.766	217.705	-143.939 (-66.11)	215.305	351.709	-136.404 (-38.78)
9	RD	Vit	272.637	367.559	-94.922 (-25.82)	521.494	564.346	-42.852 (-7.59)
10	RD	Vit	242.490	344.443	-101.953 (-29.59)	325.371	472.500	-147.129 (-31.13)
11	RD	Vit	209.180	243.932	-34.752 (-14.24)	261.299	398.496	-137.197 (-34.42)
12	RD (trauma) mac on	Vit	221.572	296.631	-75.059 (-25.30)	385.496	397.705	-12.209 (-3.07)
13	MH	Vit	359.912	552.568	-192.656 (-34.86)	504.180	554.531	-50.351 (-9.08)
14	RD	Vit	434.004	541.406	-107.402 (-19.83)	479.180	561.507	-82.327 (-14.66)
15	ERM	Vit	80.881	319.373	-238.492 (-74.67)	160.567	356.353	-195.786 (-54.94)
16	TRAUMA	Vit	101.052	124.189	-23.137 (-18.63)	146.400	236.711	-90.31 (-38.15)
17	GIANT TEAR	Vit	335.305	359.467	-24.162 (-6.72)	421.356	456.563	-35.207 (-7.71)
18	DIAGNOSTIC	Vit	110.039	390.344	-280.305 (-71.81)	165.232	395.156	-229.924 (-58.18)
19	ERM	Vit	99.844	135.527	-35.683 (-26.32)	145.354	160.996	-15.642 (-9.71)
20	VH	Vit	477.589	457.158	20.431 (4.46)	506.357	505.684	0.673 (0.13)
21	RD mac on	Vit	92.328	117.773	-25.445 (-21.60)	270.439	285.343	-14.904 (-5.22)
22	ERM	Vit	274.822	289.775	-14.953 (-5.16)	478.546	481.547	-3.001 (-0.62)
23	ERM	Vit	71.104	244.688	-173.584 (-70.94)	294.082	339.787	-45.705 (-13.45)
24	RD mac off	Vit	208.125	307.002	-98.877 (-32.20)	301.250	477.031	-175.781 (-36.85)
25	RD mac off	Vit	138.867	182.373	-43.506 (-23.85)	362.197	375.389	-13.192 (-3.51)
26	RD mac on	Vit	198.193	287.193	-89.000 (-30.99)	384.961	486.035	-101.074 (-20.79)

Of the 26 patients, 17 underwent vitrectomy due to retinal detachment (RD). Among them, three with macular pathology (macula off) showed reduction in FAZ size of the treated eye with differences from OCTA measurements of -84, -174 and -99 μm² compared to the fellow eye. Of the two patients with RD without macular pathology (macula on) one showed reduction in FAZ, with a difference of -44 μm², while the other one showed an increase of 20 μm². Three patients with RD and epiretinal membrane (ERM) had a reduced FAZ of -95, -174 and -144 μm². The remaining 10 patients with RD without any other pathology also demonstrated statistical significant reduction in FAZ size.

Vitrectomy performed in four patients with epiretinal membrane led to FAZ reduction, where three patients demonstrated differences of -25, -15 and -36 μm² and one showed a difference of -238 μm².

The remaining five patients who underwent vitrectomy showed the following pathologies: macular hole, retinal tear with vitreous haemorrhage, ocular trauma, vitreous hemorrhage and diagnostic vitrectomy. All demonstrated FAZ size reduction compared to the fellow eye.

The mean difference in FAZ size for the SCP was -93.77 ± 71.73 μm and for the DCP -88.87 ± 75.41 μm, both statistically significant (p=0.000). Comparing the reduction in size of the SCP to that of the DCP in μm, the difference was not statistically significant (p=0.585). In other words, the amount of reduction in μm was the same for both the SCP and DCP. In contrast, the amount of reduction as a percentage was not statistically different between the SCP (-33.84 ± 22.82) and the DCP (-22.54 ± 17.94) (p=0.000).

## Discussion

**Figure 1:**
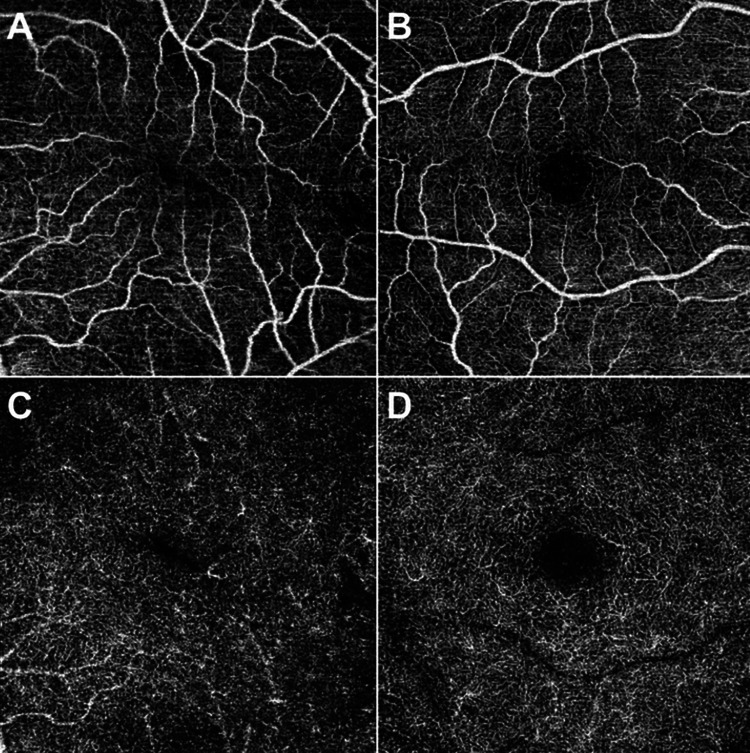
Foveal avascular zone in the operated and healthy eyes of case 15 (ERM) A: Superficial capillary plexus of the operated eye B: Superficial capillary plexus of the healthy eye C: Deep capillary plexus of the operated eye D: Deep capillary plexus of the healthy eye There is an obvious difference in the size of the avascular zone for both superficial and deep plexuses, as well as the deformation of the avascular zone. ERM: Epiretinal Membrane

In the present study, a decrease in the FAZ size (Figure 1) was demonstrated after vitrectomy for variable clinical aetiology. The observed reduction was evident even in cases where the vitrectomy did not directly influence the macular status, such as in cases of macula-on retinal detachment, vitrectomy for vitreous haemorrhage and in cases where diagnostic vitrectomy was performed.

The foveal avascular zone represents an area free of capillaries confined by interconnected capillary beds that are present in the inner retinal layers. The size of the FAZ has been shown to correlate to the age, sex and foveal thickness [10-14]. During retinal development, the FAZ is initially observed at 26 weeks gestation and remains free of capillaries until birth. It subsequently expands to reach the adult size of 500-700 μm at 15 months after birth via remodeling of the foveal pit [15]. Unless macular thickness is affected by any pathological mechanism, the size of the FAZ remains unaltered during adult life. The FAZ area is affected by ischaemic mechanisms that cause an enlargement of its size [16]. Smaller FAZ area has been associated with prematurity and ocular albinism [17,18]. Increased foveal thickness has been shown to correlate with smaller FAZ area and vice versa [19].

The role of pars plana vitrectomy in retinal oxygenation and micro-circulation has been demonstrated in the literature. After vitrectomy, the vitreous is replaced with a less viscous medium, facilitating the transport of oxygen, cytokines and other molecules. Subsequently, oxygen could reach the ischemic retinal areas and vascular endothelial growth factor (VEGF) and cytokine clearance is increased, resulting in reduction of edema and neovascularization. It is documented that oxygen alters the diameter of arterioles with direct effect on the hydrostatic balance of retinal micro-circulation. Therefore, pars plana vitrectomy affects the retinal capillary extent and function via regulation of post-operative oxygenation [2]. Improvement in perifoveal retinal micro-circulation after vitrectomy has been reported in the literature in cases with previous branch retinal vein occlusion [20].

Recently, Baba et al. [21] studied 16 eyes with macular hole that underwent vitrectomy. Optical coherence tomography angiography was performed demonstrating a significant decrease in the superficial FAZ area post-operatively. It was postulated that the centripetal movement of the foveal tissue following removal of the internal limiting membrane (ILM) may be the reason for the shallow appearance of the foveal depression and the smaller FAZ area in the post-operative cases. Similarly, Kumagai et al. [22] reported a reduction in FAZ area in one case after epiretinal membrane (ERM) and ILM peeling in a 58-year-old woman. The authors believe that the structural changes to the Müller cells following ILM removal may represent an important factor influencing the inner retinal movement causing subsequent FAZ reduction.

In our study, 26 patients with variable clinical pathology undergoing vitrectomy were included in the analysis. We included patients with and without macular pathology in order to determine whether vitrectomy alone may influence the foveal vascular micro-anatomy. A significant reduction in the area of FAZ was observed in all cases suggesting that vitrectomy may play a role in the post-operative regulation of the foveal avascular zone size. We postulate that the improvement in retinal oxygen transport following vitrectomy may influence the foveal capillary distribution and size even in cases where macular status appears normal pre-operatively.

In our cases, the reduction in FAZ size cannot be attributed (at least not solely) to ILM peeling, since peeling was performed only in cases with macular hole. The fact that there was no significant difference between the actual (absolute) size of reduction between the SCP and DCP implies that an “en-block” dislocation of both SCP and DCP vessels occurred. The difference in percentage of avascular zone reduction between the SCP and DCP was significant, further confirming that the underlying mechanism involved dislocation of SCP and DCP vessels at the same extent and most probably the same location around the avascular zone.

Furthermore, the dislocation does not appear smooth and concentric, but rather the result of “squeezing” the avascular zone along a certain axis. Another important aspect is that retinal capillaries seem to maintain their integrity during the observed dislocation (otherwise the avascular zone would appear enlarged rather than reduced). Retinal capillaries form an interconnected network of tiny vessels with minuscule length, and the maintenance of integrity requires that the feeding superficial vessels follow, if not mandate, the dislocation of the capillary plexuses.

Another important factor that should be taken into account is that during vitrectomy the viscoelastic vitreous is replaced by a non-elastic liquid. Vitreous elasticity, as well as the fact that it is firmly attached to the periphery of the retina, plays a role in the distribution of forces on the retina. This force distribution on intraocular tissues is not uniform, but rather complex given the various thickness and properties of retina, choroid and sclera in different areas of the fundus [23]. As the retina is rather loosely attached to the retinal pigment epithelium (RPE), this change may lead to a centripetal (towards macula) stretching of retinal tissue.

Although our study presents certain limitations regarding the number of cases and the inability to perform comparisons between various categories of vitrectomized eyes, it seems that the reduction in the size of the avascular zone is a universal finding. 

## Conclusions

The foveal avascular zone seems to be reduced following vitrectomy as shown by optical coherence tomography angiography. It is suggested that this may be the result of changes in the physiology of the vitrectomized eye, and that this change should be attributed to the removal of the vitreous itself rather than other structures such as the ILM. The findings if the current pilot study need further evaluation in larger scale studies to demonstrate the effects of vitrectomy in FAZ.

## References

[1] Machemer R, Buettner H, Norton EW, Parel JM (1971). Vitrectomy: a pars plana approach. Trans Am Acad Ophthalmol Otolaryngol.

[2] Stefánsson E (2009). Physiology of vitreous surgery. Graefes Arch Clin Exp Ophthalmol.

[3] Kadonosono K, Itoh N, Ohno S (2000). Perifoveal microcirculation before and after vitrectomy for diabetic cystoid macular edema. Am J Ophthalmol.

[4] Jia Y, Tan O, Tokayer J (2012). Split-spectrum amplitudedecorrelation angiography with optical coherence tomography. Opt Express.

[5] Huang Y, Zhang Q, Thorell MR (2014). Swept-source OCT angiography of the retinal vasculature using intensity differentiation-based optical microangiography algorithms. Ophthalmic Surg Lasers Imaging Retina.

[6] Jia Y, Bailey ST, Hwang TS (2015). Quantitative optical coherence tomography angiography of vascular abnormalities in the living human eye. Proc Natl Acad Sci USA.

[7] Ishibazawa A, Nagaoka T, Takahashi A (2015). Optical coherence tomography angiography in diabetic retinopathy: a prospective pilot study. Am J Ophthalmol.

[8] Kuehlewein L, Bansal M, Lenis TL (2015). Optical coherence tomography angiography of Type 1 neovascularization in agerelated macular degeneration. Am J Ophthalmol.

[9] Baba T, Kakisu M, Nizawa T, Oshitari T, Yamamoto S (2020). Regional densities of retinal capillaries and retinal sensitivities after macular hole surgery with internal limiting membrane peeling. Retina.

[10] Yu J, Jiang C, Wang X (2015). Macular perfusion in healthy Chinese: an optical coherence tomography angiogram study. Invest Ophthalmol Vis Sci.

[11] Laatikainen L, Larinkari J (1977). Capillary-free area of the fovea with advancing age. Invest Ophthalmol Vis Sci.

[12] Grunwald JE, Piltz J, Patel N, Bose S, Riva CE (1993). Effect of aging on retinal macular microcirculation: a blue field simulation study. Invest Ophthalmol Vis Sci.

[13] Gong D, Zou X, Zhang X, Yu W, Qu Y, Dong F (2016). The influence of age and central foveal thickness on foveal zone size in healthy people. Ophthalmic Surg Lasers Imaging Retina.

[14] Chui TYP, VanNasdale DA, Elsner AE, Burns SA (2014). The association between the foveal avascular zone and retinal thickness. Invest Ophthalmol Vis Sci.

[15] Provis JM, Hendrickson AE (2008). The foveal avascular region of developing human retina. Arch Ophthalmol.

[16] Conrath J, Giorgi R, Raccah D, Ridings B (2005). Foveal avascular zone in diabetic retinopathy: quantitative vs qualitative assessment. Eye.

[17] Mintz-Hittner HA, Knight-Nanan DM, Satriano DR, Kretzer FL (1999). A small foveal avascular zone may be an historic mark of prematurity. Ophthalmology.

[18] Dubis AM, Hansen BR, Cooper RF, Beringer J, Dubra A, Carroll J (2012). Relationship between the foveal avascular zone and foveal pit morphology. Invest Ophthalmol Vis Sci.

[19] Samara WA, Say EA, Khoo CT, Higgins TP, Magrath G, Ferenczy S, Shields CL (2015). Correlation of foveal avascular zone size with foveal morphology in normal eyes using optical coherence tomography angiography. Retina.

[20] Noma H, Funatsu H, Sakata K, Mimura T, Hori S (2010). Macular microcirculation before and after vitrectomy for macular edema with branch retinal vein occlusion. Graefes Arch Clin Exp Ophthalmol.

[21] Baba T, Kakisu M, Nizawa T, Oshitari T, Yamamoto S (2017). Superficial foveal avascular zone determined by optical coherence tomography angiography before and after macular hole surgery. Retina.

[22] Kumagai K, Uemura A, Furukawa M, Suetsugu T, Ogino N (2017). Decrease of the foveal avascular zone area after internal limiting membrane peeling: single case study. Int Med Case Rep J.

[23] Nickerson CS (2006). The Vitreous Humor: Mechanics and Structure. Chapter 3. http://thesis.library.caltech.edu/974/3/CSN_CH3.pdf.

